# Atlanta Medical and Surgical Journal

**Published:** 1872-04

**Authors:** 


### EDITORIAL AND MISCELLANEOUS.

### ATLANTA MEDICAL AND SURGICAL JOURNAL.

### NEW VOLUME—TENTH YEAR.

For certain satisfactory reasons at this time, we have to state that this journal does not claim or aspire to be the organ of the medical profession in the South, or of any organization, institution, or individual. It does not claim to have a larger circulation than any other medical journal, either actually or for the time of its existence, as it has no means of determining either, and is not disposed to indulge in clap-trap or misrepresentation, even if thereby its circulation could be increased. It does, however, profess a desire to be a respectable medical journal, in which the name of any medical gentleman might appear without being thereby discredited, and hopes to command the confidence of the profession everywhere, and *claims* only the intention to be a permanent journal, without regard to additional patronage, yet hopes and believes that this will be constantly increased.

As long since announced, no hope of success in connection with this journal is based upon the desire or expectation of failure upon the part of others who are honestly engaged in the same labor. We feel that there is more than space for all, and it will afford us unqualified pleasure to know that all legitimate medical journals in the South, or in any other portion of our widely-extended country, are being well sustained. We are also free to admit that there are other journals with superior advantages to our own, and better deserving the patronage of the profession in many particulars; and yet we think there can be no doubt that this journal is at least worth the subscription to any physician, however eminent or possessed of whatever advantages he may be, and is, in some considerable and satisfactory degree, meeting the *special* wants of a certain portion of the profession; and we are sure that its merits will not be enhanced, and we are determined that its success shall not be increased, by silly, baseless and empirical boastings, or the publication of fulsome and unprofessional compliments contained in communications, whether real or mythical.

